# Implantable collamer lens implantation (ICL) versus small incision lenticule extraction (SMILE) in low to moderate myopia: study protocol for a randomized, non-inferiority trial

**DOI:** 10.1186/s13063-022-06851-3

**Published:** 2022-10-28

**Authors:** Kangjun Li, Zheng Wang, Ming X. Wang

**Affiliations:** 1 The AIER School of Ophthalmology of Central, South University, Hunan Province, Changsha, China; 2 The AIER Eye and Refractive Institute of Central, South University, Hunan Province, Changsha, China; 3 Xi’an AIER Eye Hospital, Shaan’xi Province, Xi’an, 710000 China; 4 Wang Vision Institute and Aier-USA, 1801 West End Ave, Ste 1150, Nashville, TN USA

**Keywords:** Myopia, Refractive surgery, Implantable collamer lens implantation, Small incision lenticule extraction

## Abstract

**Background:**

Implantable collamer lens implantation (ICL) is a form of ‘foldable’ posterior chamber phakic intraocular lens refractive surgery that generally does not impair cornea and natural accommodation. The potential advantages of the ICL over keratorefractive laser procedures include less induction of higher-order aberrations (HOAs) and enhanced retinal image magnification. On the other hand, small incision lenticule extraction (SMILE), currently, one of the most popular refractive surgery procedures, also offers excellent visual outcomes, particularly for eyes with low to moderate amounts of myopia. The aim of this study is to evaluate whether ICL/TICL (toric ICL) is comparable to SMILE for low to moderate myopia in terms of refractive outcomes at 3 and 18 months post-operatively.

**Methods/design:**

This is a prospective randomized study. A total of 300 participants will be randomized into two groups, the ICL/TICL group and SMILE group. Eligible participants with spherical equivalent (SE) less than − 6.0 diopter (D) will be recruited. Following randomization, participants will be followed at 1, 3, 6, 12, and 18 months. The primary outcome is the refractive predictability at every postoperative point after surgery, which is the proportion of the number of eyes achieving a postoperative SE within ± 0.5 D and ± 1.0 D of the intended target. Secondary outcome parameters include visual acuity, refraction, adverse events, and quality of vision measurements.

**Discussion:**

This trial will provide information on whether ICL has comparable, if not superior, refractive outcomes compared to the established SMILE for low to moderate myopia, thus providing evidence for translation into clinical practice.

**Trial registration:**

Chinese clinical trial registry (ChiCTR) 2200055372. Registered on 08 January 2022.

## Background

Myopia, a common form of refractive error, is a leading cause of visual impairment and has been successfully corrected with refractive surgery globally [1, 2]. As refractive surgery has evolved beyond traditional laser procedures alone over the past decade, a broader surgical selection is now available, including now phakic intraocular lens [2, 3]. Currently, small incision lenticule extraction (SMILE), a form of refractive surgery, has become a popular laser refractive surgery and offers good visual outcomes, less iatrogenic dry eye, and good safety profile [4, 5]. Characterized by flapless and minimally invasive technique, SMILE has the potential for better corneal biomechanical stability [6], larger functional optical zones [7], and fewer surgically induced corneal higher-order aberrations (HOAs) [8].

The EVO Visian Implantable Collamer Lens (V4c ICL; STAAR Surgical, Monrovia, CA, USA), a posterior chamber phakic intraocular lens which incorporates CentraFLOW technology, has become widely accepted as a long-term effective approach for myopia correction, especially in the high range of myopia [9–11]. The potential advantages of the ICL over keratorefractive laser procedures include higher contrast sensitivity, higher retinal image magnification, and less induction of higher-order aberrations (HOAs) [11–13]. However, since ICL still has a potential complication related to endothelial cell loss, aqueous flare, and crystalline lens transmittance decreased in short term [14], the efficacy of ICL for low- to moderate-range myopia has not been demonstrated.

Although ICL is rapidly gaining popularity in many parts of Asia and the European Union, the number of ICL cases is still far fewer than excimer laser refractive procedures, especially in myopia with less than 6.0 diopters (D). Our previous clinical outcomes already indicated that ICL can offer acceptable safety, predictability, and stability for high myopia [15], and some comparative studies between keratorefractive laser surgery and ICL implantation for correction of high myopia concordantly showed superior refractive accuracy and subjective visual quality for the latter technique [12, 13, 16]. However, there are currently few randomized controlled trials comparing between ICL and SMILE in low to moderate myopia.

Non-inferiority trials are used to compare the standard procedure with a relatively new treatment which is expected to have some advantages such as greater predictability, safety, efficacy, and less side effects [17, 18]. SMILE was considered as the current standard refractive surgery for low to moderate myopia [2, 4] and produced good visual outcomes with refractive predictability [5]. As we do not expect to see a great improvement to the results from the already established SMILE, we aim to demonstrate that ICL is just as good in terms of visual outcome in this randomized non-inferiority trial for the low to moderate range of myopia.

## Methods/design

### Study design and randomization

**Fig. 1:**
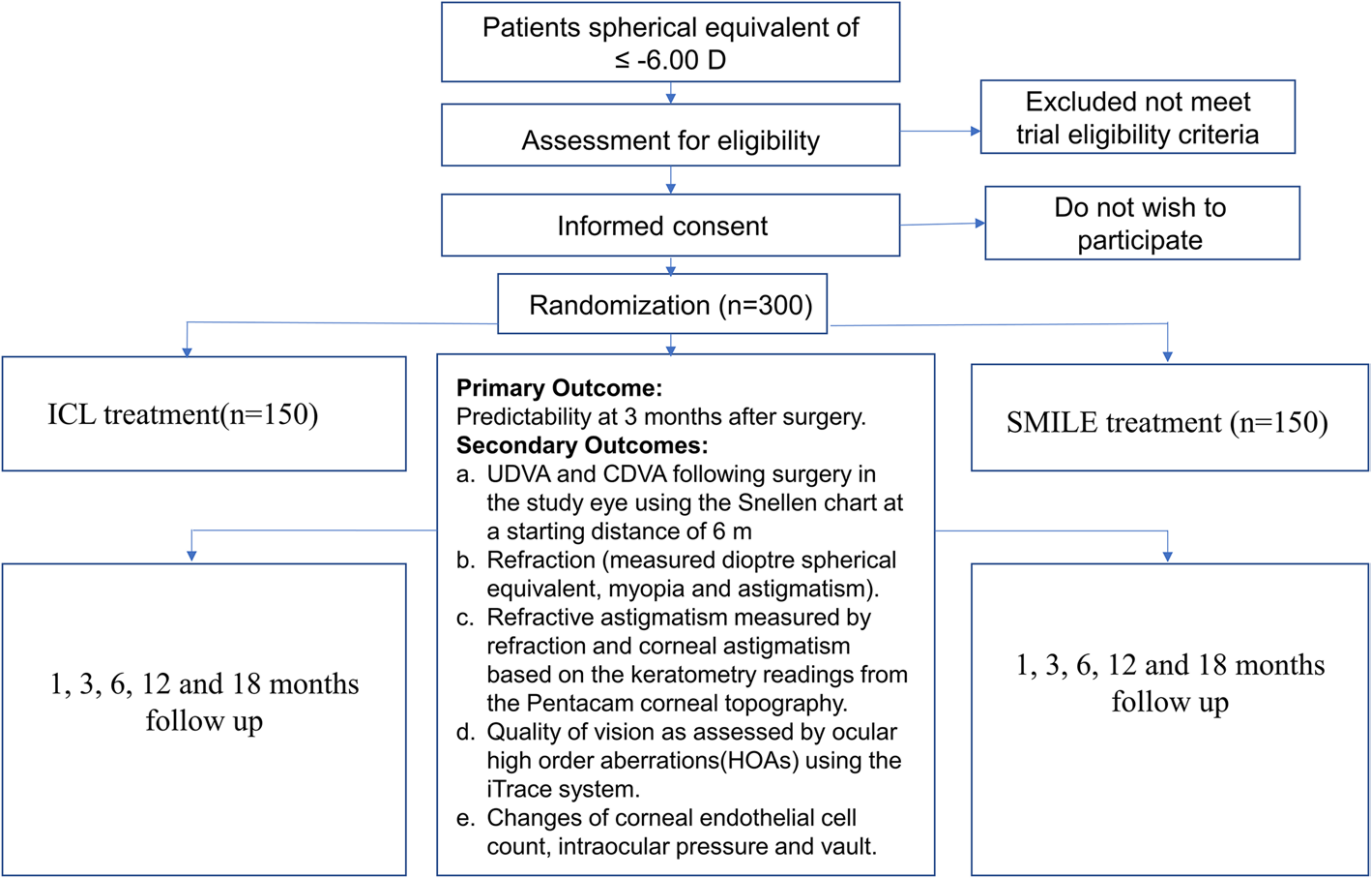
The comparing trial Consolidated Standards of Reporting Trials (CONSORT) flow diagram

**Fig. 2:**
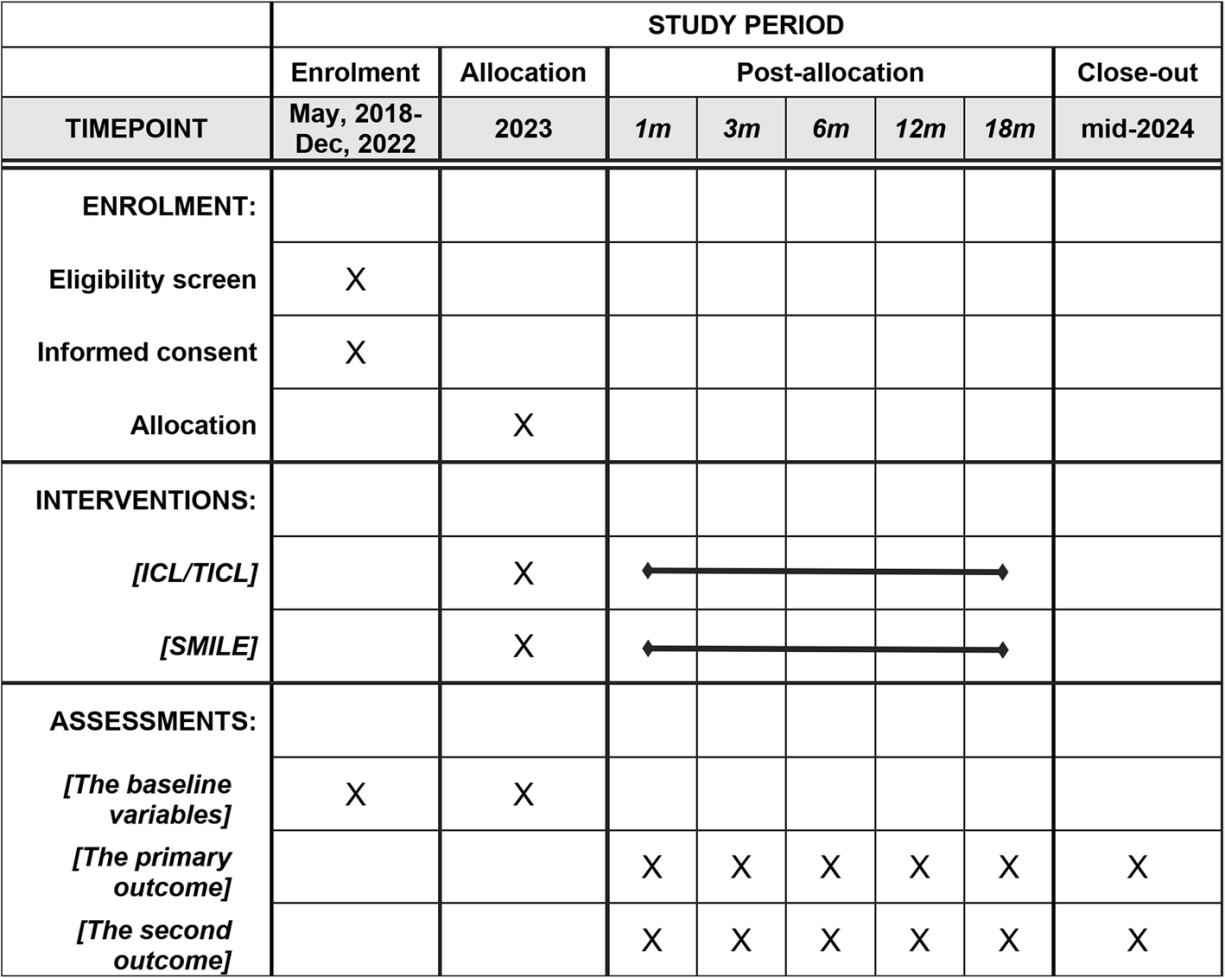
The schedule of enrolment, interventions, and assessments of this trial (SPIRIT figure)

This is a prospective, randomized controlled trial performed at Xi’An AIER eye hospital. The study adheres to the tenets of the Declaration of Helsinki and is registered at the Chinese clinical trial registry (ChiCTR2200055372) and used the SPIRIT reporting guidelines [19]. It was approved by the Xi’An AIER eye hospital ethics committee (AIER-Xian-2018001). Inclusion and exclusion criteria are shown in Table 1. Randomization will be performed on the day of surgery using a web-based, online, sealed envelope-based system (https://www.sealedenvelope.com). Specific study information sheets will be provided to patients prior to taking consent. Following a dedicated screening and randomization visit for eligible patients, participants will be randomized to one of two trial arms (Fig. 1) and then followed for 18 months at 1, 3, 6, 12, and 18 months (Fig. 2). Face-to-face adherence reminder sessions will take place at the initial trial and subsequent sessions will occur at the follow-up visits. To enhance adherence, simple strategies will be used including mobile phone or WeChat reminder service for participants at follow-up time. Because of the nature of the intervention, surgeon and participant masking will not be possible, so follow-up measurements will be performed by masked optometrists. The retreatment (secondary surgery) for refractive regression will not be permitted for all participants during the trial. However, after 18 months, retreatment must be performed for patients after refraction is stabilized. This protocol is the second version and is revised on Jan 2022. Recruitment commenced on May 2018, and the follow-up of the last recruited patient will be estimated to complete on Dec 2022.

**Table 1:** Inclusion and exclusion criteria for trial participants

**Inclusion criteria**	21 years of age or older
	Spherical equivalent of ≤ -6.00 D
	Refractive cylinder ≤ -2.00 D
	Best spectacle-corrected visual acuity (BCVA) of 20/40 or better
	Spherical or cylindrical error has progressed at − 0.50 D or less per year before the baseline measurement
	Contact lens must have been removed at least 2 weeks before the baseline measurement
	No evidence of irregular astigmatism on corneal tomography
	Anterior chamber depth ≥ 2.8 mm
	Corneal endothelial cell count ≥ 2000/mm^2^
**Exclusion criteria**	Progressive or unstable myopia and/or astigmatism
	Clinical or corneal topographic evidence of keratoconus
	Residual, recurrent or active ocular disease and retinal disease
	Previous corneal surgery
	Taking systemic medications and systemically immunocompromised or systemic disease likely to affect wound healing, such as diabetes, connective tissue disease, and severe atopy; pregnant or nursing

### Baseline assessment

At baseline all patients were assessed as follows:

1. Visual acuity (unaided and corrected), Snellen Chart at a starting distance of 6 m (m) in both eyes.
2. Subjective refraction, both eyes.
3. Corneal tomography and corneal center thickness, Scheimpflug imaging (Pentacam) in both eyes.
4. High-order aberration, ray-trace imaging (iTrace) in both eyes.

### Surgical interventions

#### ICL/TICL procedure

In the ICL/TICL group, ICL power calculation is performed by the manufacturer in all cases using the proprietary online form (https://evo-ocos.staarag.ch; version 4.08). ICL/TICL size was selected based on anterior chamber depth (Pentacam) and horizontal corneal diameter (Pentacam). After cycloplegia and topical anesthesia are administered, a model V4c ICL/TICL is inserted through a 2.8-mm clear corneal incision at the steepest meridian and the remaining ophthalmic viscosurgical device is completely washed out of the anterior chamber with a balanced salt solution. Postoperatively, nonsteroidal anti-inflammatory drugs (Pranoprofen; Pranopulin, Senju) and antibiotic medications (Levofloxacin; Cravit, Santen) are administered topically 4 times daily for 2 weeks, and the dose is steadily reduced thereafter.

#### SMILE procedure

In the SMILE group, the VisuMax 500-kHz femtosecond laser (Visumax, Carl Zeiss Meditec AG) is used to create the femtosecond laser dissection planes for SMILE. The spot distance is 3 mm for lamellar cuts and 2 mm for side cuts. The spot energy is set to 140 to 150 nJ. The minimum lenticule side-cut thickness is set to 10 mm. The cap diameter is 7.5 mm with a 2.5-mm side-cut and a side-cut angle of 90°. After surgery, patients receive levofloxacin 0.5% eyedrops (Cravit; Santen, Osaka, Japan) and dexamethasone 0.1% eyedrops (Maxidex; Alcon-Couvreur, Puurs, Belgium) 4 times daily for 2 weeks. Artificial tears (HYCOSAN 0.1%; URSAPHARM Arzneimittel GmbH, Saarbrücken, Germany) are prescribed after surgery, and the dosage is adjusted based on the patients’ symptoms.

### Outcomes and trial duration

All patients are assessed at baseline, 1, 3, 6, 12, and 18 months. We plan to use standard primary and secondary outcomes measures at 3 months postoperatively, which is reported as standard outcomes in refractive studies. The primary outcome is the refractive predictability at each time point after surgery, which is the proportion of the number of eyes achieving a postoperative spherical equivalent (SE) within ± 0.5 D and ± 1.0 D of the intended target [5]. Secondary outcomes included.

1. Unaided visual acuity (UDVA) and best corrected visual acuity (CDVA) following surgery in the study eye using the Snellen chart at a starting distance of 6 m.
2. Refraction (measured dioptric spherical equivalent, myopia, and astigmatism).
3. Refractive astigmatism measured by refraction and corneal astigmatism based on the keratometry readings from the Pentacam corneal topography.
4. Quality of vision as assessed by ocular high-order aberrations (HOAs) using the iTrace system.
5. Changes in corneal endothelial cell count, intraocular pressure, and lens vault.

### Sample size

As this is a non-inferiority trial with a binary outcome, we have calculated the required sample size using the maximum likelihood method for a large sample [20]. A review of the current literature reveals that the reported refractive predictabilities in ICL and SMILE range from 90.0% [12] to 97% [15] and from 93% [4] to 99% [5], respectively. We therefore assumed the refractive predictabilities in ICL and SMILE in this study are 95% and 97%, respectively. Thus, a sample size of 200 subjects (400 eyes) was deemed to be sufficient to confirm non-inferiority with a power of ≥ 80% and at a 5% significance level using a 2% non-inferiority margin, which is the clinically significant difference from our preliminary data. To account for a lost to follow-up rate of 30%, 300 subjects will be recruited instead of 200. Patients will be recruited for this trial through two primary mechanisms: local advertising and research patient database. Local advertising takes advantage of the audio-visual media as well as internet social platform. The Xi’an AIER refractive error patient registry (database) has been maintained since 2017 and currently contains more than ten thousand patients (more than 12 million people now live in the city).

### Data collection

Patients were first involved in this research at a patient event hosted by Xi’an AIER Eye Hospital. Topics on which opinions were collected included randomization, cross-over, and the duration of follow-up of trial patients. The staff members from Xi’an AIER Eye Hospital refractive center were responsible for generating the allocation sequence and enrolling participants. The trained research nurses, supervised by the surgeon, will obtain written consent from participants in the trial. The investigators will communicate a summary of the trial results to participants. The burden of the intervention was discussed at our initial meeting with patients and at the consent-taking stage in the trial. All patients will have data collection forms outlining the schedule of each follow-up visit and data to be collected at each visit, which include visual acuity, refraction results, clinical examination, and other outcome measures as described above. According to our previous experience and strategies for adherence, few patients will discontinue or deviate from the protocol. However, participants may withdraw from the study for any reason at any time. The primary and secondary outcomes will be collected from patients discontinued from the study at the last follow-up visit. Furthermore, the effect that any missing data might have on results will be assessed via sensitivity analysis. Dropouts (participants who non-adherence or discontinue from the protocol) will be included in the analysis by modern imputation methods for missing data. All data access and trial conduct will be monitored by the supervisors (Z.W and M.X.W). An interim analysis will be performed on the primary endpoint when 50% of patients have been randomized and have completed the 3 months follow-up. The interim analysis will be performed by an independent statistician who is blinded for the treatment allocation. The statistician will report to the AIER central ethics committee (ACEC). The ACEC will have access to all data and will discuss the results of the interim analysis. The ACEC will decide on the continuation or stopping of the trial according to interim-analysis results. At the end of the study, the research data will be entered by the research assistant and stored for up to 3 years in compliance with any integrity issues that may arise from any subsequent publications. Following that time period, the data will be kept under the control of the supervisor. The technical appendix, statistical code, and dataset are available from the Dryad repository, https://doi.org/10.5061/dryad.0vt4b8h1g.

### Adverse events

Patients are assessed for adverse events during surgery and at all postoperative visits following randomization.

1. Frequency of intraoperative events: for ICL/TICL, adverse events include lens impairment, ICL flip, iris prolapse, and hyphema; for SMILE, adverse events include suction loss, opaque bubble layer (OBL), black spots, lenticule remnants, and decentration.
2. Frequency of postoperative events: for ICL, we document the frequency of adverse events such as ocular hypertension, transient corneal edema, corneal endodermis damage, vault abnormality, surgery-related cataract, and intraocular infection; for SMILE, we document the frequency of adverse events such as infectious keratitis, diffuse lamellar keratitis (DLK), transient light sensitivity syndrome (TLSS), surgery related-cornea ectasia, and refractive regression.

All adverse events will be reported to both the centralized institution review board and institution heads (AIER Eye Group). The AIER Eye Group has insurance to cover for non-negligent harm associated with the trial. This will include coverage for additional health care or compensation according to study insurance policies.

### Statistical analyses

Demographic and baseline information will be described, and eye-specific characteristics will be described for either arm. To study the non-inferiority of ICL to SMILE, a 90% confidence interval (CI) of the difference in predictability between the two treatments (SMILE minus ICL) using a linear mixed model. If the upper limit of the 90% CI does not exceed the pre-defined non-inferiority margin of 2%, non-inferiority is confirmed. Similarly, for each of the two secondary outcomes, efficacy, and safety, a 90% CI of the difference between the two treatments using the above-mentioned method will be constructed and then compared with a non-inferiority margin of 2%. Assuming the other secondary outcome, HOA, follows a normal distribution, a 90% CI of the difference between the two treatments will be constructed through the linear mixed model, and then compared with a non-inferiority margin of 2%. All the statistical analyses of the data will be performed using SPSS 23.0 (Inc, Chicago, IL) package. Normality of data will be examined by histogram frequency analysis and the Shapiro–Wilk test. Data will be presented as the mean ± standard deviation (SD) for continuous variables. Student’s *t*-test and ANOVA for normally distributed variables or Mann–Whitney *U* test for skewed distributions will be used to compare differences, while the proportion for categorical data and chi-squared or Fisher’s exact test to test statistical significance. All statistical tests used a 2-sided *P* value of 0.05.

## Discussion

In this non-inferiority trial, we aim to demonstrate that ICL/TICL is just as good as SMILE in terms of refractive outcome, as we do not expect to see a great improvement to the results from the already established SMILE procedure for low to moderate myopia. Moreover, this trial may show that ICL/TICL may be an alternative surgical option for such eyes with subclinical keratoconus, suspect keratoconus and thin corneas (central corneal thickness [CCT] < 480 μm). On the other hand, if we use a superiority trial design with a small sample size that fails to demonstrate any difference between ICL/TICL and SMILE, would be inconclusive since it does not necessarily prove equivalence. Thus, we use a non-inferiority trial design to compare our primary and secondary outcomes [21].

Despite its proven efficacy, SMILE still requires corneal stromal tissue removed, irreversible tissue alterations could affect corneal biomechanical properties thus leading to iatrogenic keratectasia possibly [22]. So, thin cornea is one of the risk factors of iatrogenic ectasia and a minimum corneal thickness (480 μm) was always accepted by the surgeons for laser techniques [23]. For these reasons, ICL/TICL is potentially a new, improved form of refractive surgery, which may supersede SMILE and change clinical practice for thin corneas (CCT < 480 μm). Moreover, the needs for enhancements or retreatment are higher in patients with a laser surgery history, a common condition in China. ICL/TICL can be used to such patients without the extra costs involved in visual rehabilitation.

More importantly, though refractive surgery may be contraindicated in keratoconus eyes or eyes which are suspect keratoconus based on tomographic criteria, intraocular procedures such as implantable contact lenses could offer a safe and efficacious way to correct the refractive error, sparing the cornea. Hence, to be able to safely and efficaciously offer intraocular myopia-correcting procedures such as ICL for these keratoconus eyes, particularly suspect keratoconus eyes in which the keratoconus disease itself is very mild and exists only topographically (i.e., without affecting a patient’s vision), will expand the range of tools available for refractive surgeons to help these patients improving their uncorrected vision and increase their independence on glasses and contacts [15, 24]. We have previously reported the clinical experience for subclinical keratoconus corneal biomechanical characteristics and have found that ICL/TICL offered predictable refractive results [15]. We also noted that at 2-year after surgery, patients reported stable corneal biomechanics and there was no severe complication at the follow-up time [15]. These results are important when counseling patients before surgery and explaining what to expect after surgery. We have identified avenues for further research to improve early detection and stratification of patients for early identification to avoid potential iatrogenic corneal ectasia.

In conclusion, this non-inferiority clinical trial that compares ICL/TICL and SMILE will help to determine if this refractive procedure, ICL/TICL, has equal or better visual and refractive outcomes compare to the traditional SMILE for treatment of low to moderate myopia. Results of this trial will likely impact clinical practice with potentially further development into novel techniques for thin cornea and suspect keratoconus.

## Trial status

Ongoing. This protocol is the second version and is revised on Jan 2022. Recruitment commenced on May 2018, and follow-up of the last recruited patient is estimated to complete on Dec 2022.

## Abbreviations

ICL/TICL: Implantable collamer lens implantation/toric implantable collamer lens implantation
SMILE: Small incision lenticule extraction
